# Supplementary material for: Acetylcholinesterase electrochemical biosensors with graphene-transition metal carbides nanocomposites modified for detection of organophosphate pesticides
Source: PLoS One. 2020 Apr 29;15(4):e0231981. doi: 10.1371/journal.pone.0231981 (PMC7190139; doi:10.1371/journal.pone.0231981)
Supplement: S1 Fig — The overall atomic% of Ti 2p, C 1s, O 1s and F 1s are 22.22%, 29.87%, 19.19% and 28.72%. (DOCX) [file pone.0231981.s001.docx]

**1. XPS spectra**





Fig S-1 XPS spectrum of Ti_3_C_2_T_x_ nanosheets. The overall atomic% of Ti 2p, C 1s, O 1s and F 1s are 22.22%, 29.87%, 19.19% and 28.72%.
