# Supplementary material for: Acetylcholinesterase electrochemical biosensors with graphene-transition metal carbides nanocomposites modified for detection of organophosphate pesticides
Source: PLoS One. 2020 Apr 29;15(4):e0231981. doi: 10.1371/journal.pone.0231981 (PMC7190139; doi:10.1371/journal.pone.0231981)
Supplement: S2 Fig — XPS spectrum of (A) Ti3AlC2 nanosheets, (B) Ti 2p and (C) O 1s. The overall atomic% of Ti 2p, C 1s, O 1s, F 1s and Al 2p are 15.04%, 32.24%, 28.06%, 13.02% and 11.63%. Binding energy values of each bond associated with deconvoluted peaks are listed in S1 Table. (DOCX) [file pone.0231981.s002.docx]

Fig S-2 XPS spectrum of (A) Ti_3_AlC_2_ nanosheets, (B) Ti 2p and (C) O 1s. The overall atomic% of Ti 2p, C 1s, O 1s, F 1s and Al 2p are 15.04%, 32.24%, 28.06%, 13.02% and 11.63%. Binding energy values of each bond associated with deconvoluted

peaks are listed in Table S-1.
