# Supplementary material for: Acetylcholinesterase electrochemical biosensors with graphene-transition metal carbides nanocomposites modified for detection of organophosphate pesticides
Source: PLoS One. 2020 Apr 29;15(4):e0231981. doi: 10.1371/journal.pone.0231981 (PMC7190139; doi:10.1371/journal.pone.0231981)
Supplement: S3 Fig — The DPV peak current of each biosensor is 4.210, 4.139, 4.271, 4.012 and 4.085 μA. And the RSD of the DPV results is 2.491%. (DOCX) [file pone.0231981.s003.docx]

**2 Repeatability and stability results.**

**

**

Fig S-3 DPV results of five AChE/Ti_3_C_2_T_x_-CS/GR/GCE biosensors which were prepared under same production parameters. The DPV peak current of each biosensor is 4.210, 4.139, 4.271, 4.012 and 4.085 µA. And the RSD of the DPV results is 2.491%.
