# Supplementary material for: Acetylcholinesterase electrochemical biosensors with graphene-transition metal carbides nanocomposites modified for detection of organophosphate pesticides
Source: PLoS One. 2020 Apr 29;15(4):e0231981. doi: 10.1371/journal.pone.0231981 (PMC7190139; doi:10.1371/journal.pone.0231981)
Supplement: S4 Fig — After 0, 20, 30, and 40 days, the peak current of the DPV of the biosensor were 4.270, 4.092, 4.067 and 4.052 μA, respectively. (DOCX) [file pone.0231981.s004.docx]

Fig S-4 DPV results of AChE/Ti_3_C_2_T_x_-CS/GR/GCE biosensor, which was stored for 0, 20, 30, and 40 days at room temperature in PBS solution. After 0, 20, 30, and 40 days, the peak current of the DPV of the biosensor were 4.270, 4.092, 4.067 and 4.052 µA, respectively.
