# Supplementary material for: Acetylcholinesterase electrochemical biosensors with graphene-transition metal carbides nanocomposites modified for detection of organophosphate pesticides
Source: PLoS One. 2020 Apr 29;15(4):e0231981. doi: 10.1371/journal.pone.0231981 (PMC7190139; doi:10.1371/journal.pone.0231981)
Supplement: S1 Table — (DOCX) [file pone.0231981.s005.docx]

Table S-1 XPS peak fitting results for crumpled Ti_3_AlC_2_.

| Element | Component name | Component atomic% | BE (eV) | FWHM (eV) |
| --- | --- | --- | --- | --- |
| Ti 2p_3/2_  (2p_1/2_) | Ti-C | 13.41 | 454.5 (461.2) | 0.91 (1.51) |
|  | Ti^2+^ | 26.10 | 454.9 (462.3) | 1.54 (1.21) |
|  | Ti^3+^ | 20.76 | 456.3 (463.2) | 2.55 (2.60) |
|  | TiO_2_ | 24.66 | 459.1 (464.8) | 1.46 (1.83) |
|  | C-Ti-F_x_ | 15.07 | 460.4 (465.9) | 1.48 (1.37) |
| O 1s | TiO_2_ | 15.96 | 529.9 | 1.27 |
|  | C-Ti-O_x_ | 20.17 | 530.5 | 1.11 |
|  | C-Ti-(OH)_x_ | 26.57 | 531.3 | 1.33 |
|  | Al_2_O_3_ | 24.74 | 532.2 | 1.45 |
|  | H_2_O^b^ | 12.56 | 533.3 | 1.78 |
