# Supplementary material for: Acetylcholinesterase electrochemical biosensors with graphene-transition metal carbides nanocomposites modified for detection of organophosphate pesticides
Source: PLoS One. 2020 Apr 29;15(4):e0231981. doi: 10.1371/journal.pone.0231981 (PMC7190139; doi:10.1371/journal.pone.0231981)
Supplement: Attachment — Submitted filename: response to reviewers.docx [file pone.0231981.s006.docx]

Dear editor and reviewers,

Thank you very much for your email with which you sent us the reviewer’s report on our paper with reference number PONE-D-19-31451. We also wish to take this opportunity to thank the reviewer for the constructive comments and valuable recommendations. We have carefully revised the manuscript according to the reviewer’s suggestion.

At last, please allow us to express our appreciation to your observations and suggestions for improvement of our article. I sincerely hope that the revised manuscript is now suitable for publication, if still not, one more chance to improve the quality of manuscript will be greatly appreciated.

Yours Sincerely,

Lianqiao Yang

**Responses to Editor**

Our responses to the comments and questions of editor are listed below and the corresponding revisions are marked in red in the revised manuscript.

**Question 1: The role of metal carbide should be made clearer in the modification of graphene-based biosensor**

**Reply:** Dear editor, thank you very much for your suggestions and we have made changes to this section.

In this study, the roles of Ti_3_C_2_T_x_ are: first, Ti_3_C_2_T_x_ is a hydrophilic, biocompatible nanomaterial, which can effectively improve the efficiency of enzyme fixation. Second, Ti_3_C_2_T_x_ has a large specific surface area and conductivity, which can improve the electrical performance of the electrode.

The revised part is as follows:

“In this article, dichlorvos (DDVP) was used as the representative of OPs, and GR was used as the electrode modification material, Ti_3_C_2_T_x_-CS was used as the immobilization matrix of the enzyme and an electrochemical biosensor was fabricated with structure of AChE/Ti_3_C_2_T_x_-CS/GR/glassy carbon electrode (GCE). Here, Ti_3_C_2_T_x_ has two functions. First of all, Ti_3_C_2_T_x_ is a hydrophilic, biocompatible nanomaterial, which can effectively improve the efficiency of enzyme fixation [23]. Secondly, Ti_3_C_2_T_x_ has a large specific surface area and conductivity, which can improve the electrical performance of the electrode [28]. The biosensor manufacturing process was optimized to improve performance. The catalytic ability, DDVP detection and stability were characterized by biosensors. Finally, the biosensor was tested on the real sample with tap water to verify its applicability.”

**Question 2:** **Is there any effect on oxidation state of Titanium? How about the stability of** **Ti_3_C_2_T_x_ in the modified sensor?**

**Reply:** Dear editor, the stability of Ti_3_C_2_T_x_ is relatively well. First, from Table 1, the component atomic% of TiO_2_ in the titanium element analysis is 14.75%. At the same time, the analysis result of oxygen element also contains 19.70% of TiO_2_. It indicates that the oxidation state of titanium does exist. Secondly, in the XPS analysis of Ti_3_AlC_2_, some oxygen elements also exist in the form of TiO_2_. From the results of oxygen analysis in the XPS spectrum of Ti_3_AlC_2_, the peak at 529.9eV is related to TiO_2_, component atomic% is 15.96%, as shown in the Fig S-2 below. This indicates that the oxidation state of titanium in Ti_3_C_2_T_x_ may not be caused by instability, but it exists originally. Finally, the component atomic% of TiO_2_ in Table 1 is close to that of Shah et al. (See Table 1 in reference: Shah SA, Habib T, Gao H, Gao P, Sun W, Green MJ, et al. Template-free 3D titanium carbide (Ti_3_C_2_T_x_) MXene particles crumpled by capillary forces. Chem Commun (Camb). 2016;53(2):400-3. doi: 10.1039/c6cc07733a. PubMed PMID: 27958604.)

In general, Ti_3_C_2_T_x_ used in the modified sensor is relatively stable, and the oxidation state of titanium element already exists in Ti_3_AlC_2_ before preparation.

Fig S-2 XPS spectrum of (A) Ti_3_AlC_2_ nanosheets, (B) Ti 2p and (C) O 1s. The overall atomic% of Ti 2p, C 1s, O 1s, F 1s and Al 2p are 15.04%, 32.24%, 28.06%, 13.02% and 11.63%. Binding energy values of each bond associated with deconvoluted

peaks are listed in Table S-1.

Table S-1 XPS peak fitting results for crumpled Ti3AlC2.

| Element | Component name | Component atomic% | BE (eV) | FWHM (eV) |
| --- | --- | --- | --- | --- |
| Ti 2p_3/2_  (2p_1/2_) | Ti-C | 13.41 | 454.5 (461.2) | 0.91 (1.51) |
|  | Ti^2+^ | 26.10 | 454.9 (462.3) | 1.54 (1.21) |
|  | Ti^3+^ | 20.76 | 456.3 (463.2) | 2.55 (2.60) |
|  | TiO_2_ | 24.66 | 459.1 (464.8) | 1.46 (1.83) |
|  | C-Ti-F_x_ | 15.07 | 460.4 (465.9) | 1.48 (1.37) |
| O 1s | TiO_2_ | 15.96 | 529.9 | 1.27 |
|  | C-Ti-O_x_ | 20.17 | 530.5 | 1.11 |
|  | C-Ti-(OH)_x_ | 26.57 | 531.3 | 1.33 |
|  | Al_2_O_3_ | 24.74 | 532.2 | 1.45 |
|  | H_2_O^b^ | 12.56 | 533.3 | 1.78 |

Table 1. XPS peak fitting results for crumpled Ti_3_C_2_T_x_.

| Element | Overall atomic% | Component name | Component atomic% | BE (eV) | FWHM (eV) |
| --- | --- | --- | --- | --- | --- |
| Ti 2p_3/2_  (2p_1/2_) | 22.22 | Ti-C | 24.32 | 454.9 (461.2) | 0.87 (1.45) |
|  |  | Ti^2+^ | 27.32 | 455.7 (462.4) | 1.36 (1.76) |
|  |  | Ti^3+^ | 24.16 | 456.9 (463) | 1.95 (1.86) |
|  |  | TiO_2_ | 14.75 | 459.6 (464.9) | 1.64 (2.23) |
|  |  | C-Ti-F_x_ | 9.45 | 460.1 (465.7) | 2.06 (1.36) |
| C 1s | 29.87 | C-Ti-T_x_^a^ | 42.14 | 281.9 | 0.78 |
|  |  | C-Ti-T_x_^a^ |  | 282.8 | 1.02 |
|  |  | C-C | 31.93 | 284.7 | 1.64 |
|  |  | CH_x_/CO | 19.56 | 286.2 | 2.55 |
|  |  | COO | 6.37 | 289.1 | 1.79 |
| O 1s | 19.19 | TiO_2_ | 19.70 | 529.8 | 0.96 |
|  |  | C-Ti-O_x_ | 15.48 | 530.5 | 1.11 |
|  |  | C-Ti-(OH)_x_ | 14.55 | 531.2 | 1.38 |
|  |  | Al_2_O_3_ | 27.55 | 532.0 | 1.55 |
|  |  | H_2_O^b^ | 22.72 | 533.4 | 2.06 |
| F 1s | 28.72 | C-Ti-F_x_ | 46.32 | 685.2 | 1.59 |
|  |  | AlF_x_ | 53.68 | 687.0 | 1.8 |

**Question 3:** **3.	In the XRD plot, X-axis should be changed to 2 theta (degree).**

**Reply:** Thank you very much for pointing it out. The XRD plot has been modified and added plot before HF etching for comparison.

Fig 3. XRD patterns of samples prepared by HF etching pristine Ti_3_AlC_2_

**Question 4:	Manuscript should be check for typo errors.**

**Reply:** Thanks for your suggestion. The language of the whole manuscript has been carefully checked and polished to make the manuscript more brief, clear and definite.

**Responses to Reviewer:1**

Our responses to the comments and questions of editor are listed below and the corresponding revisions are marked in blue in the revised manuscript.

**Comments:**

**Authors have applied GR, MXene, Chitosan and acetylcholinesterase modified biosensor for OP pesticides detection. The work seems interesting and very useful for application in real sample analysis. However, some points need to be addressed before its publication.**

**Reply:** Your recognition to our work and constructive suggestions are greatly appreciated.

**Question 1:	English needs to be improved throughout the manuscript.**

**Reply:** Dear reviewer, both the language and spelling errors in the manuscript have been carefully revised.

**Question 2:	A comparison table (updated citing recent works) should be included in the manuscript discussing the electrochemical performance of fabricated biosensor with other MXene based sensors and biosensors towards pesticides detection.**

**Reply:** Dear reviewer, thank you very much for your relevant suggestions. A recent comparison table of research on MXene-based biosensors for the detection of organophosphorus pesticides was added to the manuscript with related descriptions.

Table 3. Comparison with literature reported AChE biosensors.

| Biosensor structure | Linear range / μM | LOD / nM | Analyte | References |
| --- | --- | --- | --- | --- |
| AChE/Ti_3_C_2_T_x_-CS/GR/GCE | 11.31 to 1.83×10^-2^ | 14.45 | Dichlorvos | This work. |
| AChE/CS-Ti_3_C_2_T_x_/GCE | 10^-2^ to 10^-8^ | 3×10^-4^ | Malathion | Zhou et al.[38] |
| AChE/Ag@Ti_3_C_2_T_x_/GCE | 10^-2^ to 10^-8^ | 3.27×10^-6^ | Malathion | Jiang et al.[39] |
| AChE-Chit/MXene/Au NPs/MnO_2_/Mn_3_O_4_/GCE | 1 to 10^-6^ | 1.34×10^-4^ | Methamidophos | Song et al.[40] |

The revised part is as follows:

“At present, there are few reported studies on the application of MXene in the detection of OPs as shown in Table 3. Zhou et al. and Jiang et al. from the same laboratory have achieved good results using Ti_3_C_2_T_x_-based sensors to detect malathion. Song et al. designed a complex biosensor and achieved good results in detecting methamidophos. In this study, AChE biosensors with Ti_3_C_2_T_x_ modification also showed better performance compared to those without Ti_3_C_2_T_x_ modification. In addition, the biosensor can be stored at room temperature and exhibits excellent stability.”

41.	Stoytcheva M, Zlatev R, Velkova Z, Valdez B, Ovalle M, Petkov L. Hybrid electrochemical biosensor for organophosphorus pesticides quantification. Electrochimica Acta. 2009;54(6):1721-7. doi: 10.1016/j.electacta.2008.09.063.

42.	Zheng D, Vashist SK, Al-Rubeaan K, Luong JH, Sheu FS. Rapid and simple preparation of a reagentless glucose electrochemical biosensor. Analyst. 2012;137(16):3800-5. doi: 10.1039/c2an35128e. PubMed PMID: 22763782.

**Question 3:	Introduction should be improved. Much more should be added about MXene based sensors and its properties.**

**Reply:** Dear reviewer, thank you very much for your relevant suggestions. The introduction has been modified. We have added a description of MXene and the performance of MXene-based sensors.

The revised part is as follows:

“MXenes has been tried and applied in many fields such as bio- and gas-sensors, energy storage, electromagnetic interference shielding, reinforcement for composites, water purification, lubrication, and chemical, photo- and electro-catalysis due to their many advantages, such as large surface area, good hydrophilicity, and so on [22, 23]. In the application of electrochemical biosensors, MXenes has achieved satisfactory results. Quan et al. used 3D sodium titanate nanoribbons synthesized by MXene to modify electrodes for use in electrochemical biosensors to detect PSA [24]. Haiyuan et al. made DNA probes on MXene, prepared electrochemical immunosensors, and used them to detect the breast cancer marker Mucin1[25].Wang et al. developed an electrochemical biosensor based on DNA nanostructures and MXene to detect mycotoxins and the biosensor could achieve a low limit of detection of 5 pM in the range of 5 pM to10 nM [26].”

**Question 4:	What do the prominent peaks in XRD of MXene reveal?**

**Reply:** Dear reviewer, in order to better explain the prominent peaks in XRD spectrum, we added the XRD spectrum before etching as a comparison and modified the description of XRD results.

The revised part is as follows:

“The comparison of MXene before and after etching was probed by XRD analysis shown in Fig 3. After HF etched, the strongest diffraction peak of Ti_3_AlC_2_ in 38.84° (104) disappeared, indicating that the structure of the MAX phase (Ti_3_AlC_2_) material was completely destroyed, and most of the Al layer was etched. In addition, peaks in 9.53° (002) and 19.13° (004) shifted to small angles of 8.9° and 18.31° after the etching. Respectively, their full width at half maximum (FWHM) were larger after the HF acid etching, which should be attributed to the partial replacement of Al by OH-/F- [32, 33]. The peaks at 35.98 (103) and 41.75 (105) are still obvious, indicating that the MAX phase still exists and the etching is incomplete [34].”

Fig 3. XRD spectrum of MXene before and after etching with HF.

**Question 5:	What is** **the atomic percentage of elements in XPS analysis.**

**Reply:** Dear reviewer, the atomic percentage of elements in XPS analysis were 22.22% (Ti), 29.87% (C), 19.19% (O) and 28.72% (F). The detailed information is shown in Table 1.

Table 1. XPS peak fitting results for crumpled Ti_3_C_2_T_x_.

| Element | Overall atomic% | Component name | Component atomic% | BE (eV) | FWHM (eV) |
| --- | --- | --- | --- | --- | --- |
| Ti 2p_3/2_  (2p_1/2_) | 22.22 | Ti-C | 24.32 | 454.9 (461.2) | 0.87 (1.45) |
|  |  | Ti^2+^ | 27.32 | 455.7 (462.4) | 1.36 (1.76) |
|  |  | Ti^3+^ | 24.16 | 456.9 (463) | 1.95 (1.86) |
|  |  | TiO_2_ | 14.75 | 459.6 (464.9) | 1.64 (2.23) |
|  |  | C-Ti-F_x_ | 9.45 | 460.1 (465.7) | 2.06 (1.36) |
| C 1s | 29.87 | C-Ti-T_x_^a^ | 42.14 | 281.9 | 0.78 |
|  |  | C-Ti-T_x_^a^ |  | 282.8 | 1.02 |
|  |  | C-C | 31.93 | 284.7 | 1.64 |
|  |  | CH_x_/CO | 19.56 | 286.2 | 2.55 |
|  |  | COO | 6.37 | 289.1 | 1.79 |
| O 1s | 19.19 | TiO_2_ | 19.70 | 529.8 | 0.96 |
|  |  | C-Ti-O_x_ | 15.48 | 530.5 | 1.11 |
|  |  | C-Ti-(OH)_x_ | 14.55 | 531.2 | 1.38 |
|  |  | Al_2_O_3_ | 27.55 | 532.0 | 1.55 |
|  |  | H_2_O^b^ | 22.72 | 533.4 | 2.06 |
| F 1s | 28.72 | C-Ti-F_x_ | 46.32 | 685.2 | 1.59 |
|  |  | AlF_x_ | 53.68 | 687.0 | 1.8 |

**Question 6:	Why authors have chosen GR and MXene nanocomposite as GR is itself is very conducting material. What is the novelty of this work as numerous works have been reported about pesticide detection using MXene and GR sensors.**

**Reply:** Dear reviewers, in this paper, the good conductivity of graphene and Ti_3_C_2_T_x_ can improve the electrochemical property of glassy carbon electrodes by increasing the effective area of the electrode. In addition, the hydrophilicity of Ti_3_C_2_T_x_ makes it effectively to immobilize enzymes, which makes up for the disadvantages of graphene due to its hydrophobicity. However, Ti_3_C_2_T_x_ is not easily fixed to the electrode without help of other materials. The van der Waals force between the graphene layer and the layer allows it to adhere to the surface of the glassy carbon electrode without the help of other materials.

The novelty of this work: This article is the first report using GR stripped by NMP intercalation and Ti_3_C_2_T_x_, which have complementary advantages, to prepare biosensors and detect OPs. And the biosensor showed a good catalytic performance for acetylcholine.

**Question 7:	Abstract and conclusion should be different and should not repeat the data. I suggest to discuss experimental results only in abstract while major findings should be discussed in conclusion section.**

**Reply:** Dear reviewers, thank you very much for your relevant suggestions. Based on your suggestions, we have modified the abstract and conclusion.

The revised abstract is as follows:

“An acetylcholinesterase biosensor modified with graphene and transition metal carbides was prepared to detect organophosphorus pesticides. Cyclic voltammetry, differential pulse voltammetry, and electrochemical impedance spectroscopy were used to characterize the electrochemical catalysis of the biosensor: acetylcholinesterase/chitosan-transition metal carbides/graphene/glassy carbon electrode. With the joint modification of graphene and transition metal carbides, the biosensor has a good performance in detecting dichlorvos with a linear relationship from 11.31 µM to 22.6 nM and the limit of detection was 14.45 nM. Under the premise of parameter optimization, the biosensor showed a good catalytic performance for acetylcholine. Compared to the biosensors without modification, it expressed a better catalytic performance due to the excellent electrical properties, biocompatibility and high specific surface area of graphene, transition metal carbides. Finally, the biosensor exhibits good stability, which can be stored at room temperature for one month without significant performance degradation, and has practical potential for sample testing.”

The revised conclusion is as follows:

“Herein, An AChE electrochemical biosensor with a structure of AChE/Ti_3_C_2_T_x_-CS/GR/GCE was prepared by layer-by-layer casting. The biosensor exhibits obviously electrochemical signal amplification with GR and Ti_3_C_2_T_x_ modifying and increasing the effective area of the GCE. In addition, under the contribution of the large specific surface area of Ti_3_C_2_T_x_, the biosensor has strong electrochemical catalytic performance, in which the apparent Michaelis–Menten constant K_m_ is 4.89 mM. Under the fabrication process parameters optimized, the linear range of biosensors for DDVP detecting is from 18.1 nM to 11.31 μM and the LOD was 14.45 nM. In addition, biosensors exhibit good stability and can be stored for 40 days in a room temperature. The developed AChE biosensor shows high stability, reproducibility, sensitivity, accuracy and application potential in the process of real sample testing.”

**Question 8:	Figures should be more clear and of equal size and dimensions.**

**Reply:** Dear reviewers, thank you very much for your relevant suggestions. All figures in this manuscript have been uploaded in TIFF format with the same dimensions.

**Question 9:	Literature should be updated. pls include recent articles related to MXene sensing to increase the impact of material. For eg. TrAC Trends in Analytical Chemistry, 105 (2018) 424-435, Biosensors Bioelectronics, 107 (2018) 69-75**

**Reply:** Dear reviewers, the literature in the manuscript has been updated, based on your suggestions.

**Question 10:	How the stability of enzyme biosensor was justified.**

**Reply:** Generally, the storage condition of the enzyme biosensor is 4 °C. However, the enzyme biosensor in this study can be stored at room temperature (25 °C) for 40 days and maintain its catalytic activity. “The fabricated biosensor can be stored at room temperature immersing in PBS. It is so stable that there was still 95% of the initial catalytic current value after 40 days.” This shows that the biosensor has well stability.

**Question 11:	Have authors done selectivity analysis of biosensor in presence of different pesticides as it is an important parameter.**

**Reply:** Dear reviewer, your suggestion is very pertinent. We had meant to add selective experiments in the presence of different pesticides. However, due to the influence of COVID-19, we cannot return to the laboratory for experiments until now. In principle, AChE can be inhibited by organophosphorus pesticides containing O = P or S = P bonds. Therefore, as long as the pesticide contains organic molecules of O = P bond or S = P bond, AChE can be inhibited. In general, the biosensor has the ability to detect all organophosphorus pesticides. In addition, some articles on the detection of organophosphorus pesticides use only one organophosphorus pesticide to characterize their selectivity. We have listed some articles as follows for your reference:
1.	Jain U, Gupta S, Chauhan N. Construction of an amperometric glycated hemoglobin biosensor based on Au-Pt bimetallic nanoparticles and poly (indole-5-carboxylic acid) modified Au electrode. Int J Biol Macromol. 2017;105(Pt 1):549-55. doi: 10.1016/j.ijbiomac.2017.07.084. PubMed PMID: 28716755.

2.	Khairy M, Ayoub HA, Banks CE. Non-enzymatic electrochemical platform for parathion pesticide sensing based on nanometer-sized nickel oxide modified screen-printed electrodes. Food Chem. 2018;255:104-11. doi: 10.1016/j.foodchem.2018.02.004. PubMed PMID: 29571455.

3.	Kumar THV, Sundramoorthy AK. Electrochemical biosensor for methyl parathion based on single-walled carbon nanotube/glutaraldehyde crosslinked acetylcholinesterase-wrapped bovine serum albumin nanocomposites. Anal Chim Acta. 2019;1074:131-41. doi: 10.1016/j.aca.2019.05.011. PubMed PMID: 31159933.

4.	Lu X, Tao L, Li Y, Huang H, Gao F. A highly sensitive electrochemical platform based on the bimetallic Pd@Au nanowires network for organophosphorus pesticides detection. Sensors and Actuators B: Chemical. 2019;284:103-9. doi: 10.1016/j.snb.2018.12.125.

5.	Mishra RK, Vinu Mohan AM, Soto F, Chrostowski R, Wang J. A microneedle biosensor for minimally-invasive transdermal detection of nerve agents. Analyst. 2017;142(6):918-24. doi: 10.1039/c6an02625g. PubMed PMID: 28220163.

6.	Xie Y, Yu Y, Lu L, Ma X, Gong L, Huang X, et al. CuO nanoparticles decorated 3D graphene nanocomposite as non-enzymatic electrochemical sensing platform for malathion detection. Journal of Electroanalytical Chemistry. 2018;812:82-9. doi: 10.1016/j.jelechem.2018.01.043.

7.	Xu G, Huo D, Hou C, Zhao Y, Bao J, Yang M, et al. A regenerative and selective electrochemical aptasensor based on copper oxide nanoflowers-single walled carbon nanotubes nanocomposite for chlorpyrifos detection. Talanta. 2018;178:1046-52. doi: 10.1016/j.talanta.2017.08.086. PubMed PMID: 29136795.

8.	Zhang Y, Fa H-b, He B, Hou C-j, Huo D-q, Xia T-c, et al. Electrochemical biomimetic sensor based on oxime group-functionalized gold nanoparticles and nitrogen-doped graphene composites for highly selective and sensitive dimethoate determination. Journal of Solid State Electrochemistry. 2017;21(7):2117-28. doi: 10.1007/s10008-017-3560-0.

**Responses to Reviewer: 2**

Our responses to the comments and questions of editor are listed below and the corresponding revisions are marked in green in the revised manuscript.

**Comments:**

**This paper by Wang et.al presents the fabrication of a biosensor based on graphene-transition metal carbides nanocomposite for detection of organophosphate pesticides which are important food biomarkers. The experimental work is systematic and reasonably organized. The paper may be accepted after subject to addressing of following comments.**

**Reply:** Dear reviewer, your recognition to our work and constructive suggestions are greatly appreciated.

**Question 1:	Here are numerous English grammar, and sentence misappropriation mistakes throughout the manuscript. For instance, the caption of Fig. 1C shall be replaced with Fig. 1D and vice versa. The word “purchased” should be included in the first line in “Materials and chemicals”**

**Reply:** Dear reviewer, your suggestions are very pertinent. The grammar and sentence misappropriation mistakes in the manuscript were carefully modified. Figure 1 has been modified based on your suggestions. The “Materials and chemicals” section has also been modified.

Fig 1．The SEM image of (A) GR, (B) Ti_3_C_2_T_x_ and (C) AChE, and TEM image of (D) Ti_3_C_2_T_x_.

**Purchased materials and chemicals**

AChE (from electric eel), ATCl, and DDVP were from Sigma-Aldrich. CS (viscosity >400 mPa s), Bovine serum albumin (BSA) and GR (stripped by 1-Methyl-2-pyrrolidinone (NMP)) were from Aladdin Bio-Chem Technology (Shanghai, China). Ti_3_C_2_T_x_ was from HAOXI Research Nanomaterials. The other chemicals used in the experiments were from Sinopharm Chemical Reagent Co., Ltd (Shanghai, China). All chemical reagents used are of analytical grade. Nitrogen with a purity of 99.9% comes from Air Liquide (China) Holding Co., Ltd

**Question 2:	Fig. 2B XRD exhibits very sharp peak of (103) and (105) which correspond to MAX phase. This indicates that there is considerable unetched MAX phase, unable to convert to MXene. Please comment in the light of reference 10.1016/j.jallcom.2018.04.152, Materials Science and Engineering B 191 (2015) 33–40 etc**

**Reply:** Dear reviewer, thank you for pointing out our mistake. We have modified the description of the XRD spectrum based on suggestions from you and Reviewer 1.

The revised part is as follows:

“The comparison of MXene before and after etching was probed by XRD analysis shown in Fig 3. After HF etched, the strongest diffraction peak of Ti_3_AlC_2_ in 38.84° (104) disappeared, indicating that the structure of the MAX phase (Ti_3_AlC_2_) material was completely destroyed, and most of the Al layer was etched. In addition, peaks in 9.53° (002) and 19.13° (004) shifted to small angles of 8.9° and 18.31° after the etching. Respectively, their full width at half maximum (FWHM) were larger after the HF acid etching, which should be attributed to the partial replacement of Al by OH-/F- [32, 33]. The peaks at 35.98 (103) and 41.75 (105) are still obvious, indicating that the MAX phase still exists and the etching is incomplete [34]”

Fig 3. XRD spectrum of MXene before and after etching with HF.

**Question 3:	Line 154, page 11, please remove “and the”. Similarly correct the sentence in line 131 on page 11.**

**Reply:** Dear reviewer, thank you for pointing out the mistakes. We have corrected the errors you pointed out, and we have also revised other similar errors in the manuscript.

**Question 4:	The EIS spectra curve (c) in Fig. 3B has different behavior as compared to other three curves in the higher frequency range (other have two semi-circles whereas curve (c) has a single semi-circle. Please comment**

**Reply:** Dear reviewer, the curve (c) in the EIS spectrum does not have two semicircles. Each curve shown in the figure has a similar "semi-circle" in the higher frequency range. In the EIS test, in order to capture the semi-circle related to transmission impedance R_CT_ of the electrode, the frequency will be adjusted in a wide range. So, a semi-circle-like tail appears before the semi-circle appears as shown in below. When drawing and fitting calculations, the tail of the high frequency part is generally deleted. For the data integrity, we kept all the data in the drawing without any deletion. We also found an article with a similar small tail in the EIS spectrum.

Figure. The EIS spectrum of AChE/Ti_3_C_2_T_x_-CS/GR/GCE with a small tail.

Cui HF, Wu WW, Li MM, Song X, Lv Y, Zhang TT. A highly stable acetylcholinesterase biosensor based on chitosan-TiO2-graphene nanocomposites for detection of organophosphate pesticides. Biosens Bioelectron. 2018;99:223-9. doi: 10.1016/j.bios.2017.07.068. PubMed PMID: 28763783.

**Question 5:	Authors explain in Fig.5 that GR-modified biosensor is better in performance as compared to MXene-modified biosensor, however the synergistic enhancement occurs when biosensor is fabricated with MXene-GR-modification. What is the exact mechanism for enhancement of catalytic ability in composite biosensor?**

**Reply:** First of all, only GR-modified sensor has better performance than only MXene-modified sensor from the results. This shows that GR has a better electrical signal amplification effect. The hydrophilic MXene has better potential for immobilizing enzymes. Under the modification of MXene and GR, the biosensor performs better, which can be attributed to two reasons: firstly, the effective area of the sensor is larger with MXene-GR-modification, which can be seen from the CV characterization; Second, the hydrophilicity of MXene can make up for the lack of GR hydrophobicity.

The revised part is as follows:

“To further demonstrate the effect of different modifying materials on biosensor performance, the DPV curve with different modifications was employed in 1 mM ATCl as shown in Fig 6. It is obvious that the curve c with the structure of AChE/Ti_3_C_2_T_x_-CS/GR/GCE has the highest peak. Without GR or Ti_3_C_2_T_x_, it has a significant reduction in peaks. Among them, when there is no GR, the sensor has the weakest catalytic ability (curve b). This shows that in the sensor, GR acts as the most significant electrochemical signal amplification. Only Ti_3_C_2_T_x_ modified biosensors cannot effectively collect electrical signals due to the weak conductivity of CS. When GR and Ti_3_C_2_T_x_ are used together, complementary advantages of the electric signal amplification of GR and the hydrophilicity of Ti_3_C_2_T_x_ which is easy to immobilize enzymes make the biosensors have better performance [23, 28].”

**Question 6:	What is the reason for high recovery (%) of 109 for sample 2 in Table 1**

**Reply:** Recovery% is the ratio of the detected value to the true value, so it may exceed 100%. The meaning of Recovery% is that the closer it is to 100%, the more accurate the test result is.
